# High-Pressure Synthesis and Characterization of the Actinide Borate Phosphate U_2_[BO_4_][PO_4_]

**DOI:** 10.1002/ejic.201300662

**Published:** 2013-09-10

**Authors:** Ernst Hinteregger, Klaus Wurst, Lukas Perfler, Florian Kraus, Hubert Huppertz

**Affiliations:** [a] Institut für Allgemeine, Anorganische und Theoretische Chemie, Leopold-Franzens-Universität InnsbruckInnrain 80–82, 6020 Innsbruck, Austria; [b] Institut für Mineralogie und Petrographie, Leopold-Franzens-Universität InnsbruckInnrain 52f, 6020 Innsbruck, Austria; [c] AG Fluorchemie, Department Chemie, Technische Universität MünchenLichtenbergstr. 4, 85747 Garching, Germany

**Keywords:** Actinides, Uranium, High-pressure chemistry, Borate phosphates

## Abstract

A new actinide borate phosphate, U_2_[BO_4_][PO_4_], was synthesized in a Walker-type multianvil apparatus at 12.5 GPa and 1000 °C. The crystal structure was determined from single-crystal X-ray diffraction data collected at room temperature. U_2_[BO_4_][PO_4_] crystallizes in the monoclinic space group *P*2_1_/*c* with four formula units per unit cell and the lattice parameters *a* = 854.6(2), *b* = 775.3(2), *c* = 816.3(2) pm, and *β* = 102.52(3)°. The structure consists of double layers of linked uranium–oxygen polyhedra parallel to [100]. The borate tetrahedra are located between the uranium–oxygen layers inside the double layer. The phosphate groups link the double layers.

## Introduction

The structural chemistry of borates exhibits a respectable diversity, which comes from the ability of the boron atom to form trigonal-planar [BO_3_]^3–^ groups and tetrahedral [BO_4_]^5–^ groups. Moreover, links between these groups to form chains, layers, or highly condensed three-dimensional networks enlarge the amount of possible compounds. Phosphates show similar properties and can form isolated ions such as the diphosphate anion [P_2_O_7_]^4–^ or the triphosphate anion [P_3_O_10_]^5–^, infinite chains such as those in the mercury(II) polyphosphate Hg(PO_3_)_2_,^1^ or layers as in Ag_2_PdP_2_O_7_.^2^ Therefore, the combination of borate and phosphate groups leads to an enormous amount of possible structures. Although the ternary U–B–O system is represented by only two different oxoborates with the compositions U(BO_3_)_2_^3^ and (UO_2_)(B_2_O_4_),^4^ more than ten compounds are known in the U–P–O system [e.g., U(PO_3_)_4_,^5^ U(P_2_O_7_),^6^ and U_2_(PO_4_)(P_3_O_10_)].^7^ Interestingly, there are no quaternary uranium compounds that contain both borate and phosphate groups. In general, compounds with borate and phosphate groups can be categorized in the classes of borophosphates and borate phosphates. In borophosphates, the [BO_3_]^3–^, [BO_4_]^5–^, and [PO_4_]^3–^ groups are connected among each other. If the borate and phosphate groups are isolated, the compounds are designated as borate phosphates. In recent years, the field of borophosphate chemistry has been greatly extended. Selected examples of this class are the compounds M^II^[BPO_4_(OH)_2_] (M^II^ = Mn, Fe, Co),^8^ Mg_3_(H_2_O)_6_[BPO_4_(OH)_3_]_2_,^9^ and Ba_3_(BP_3_O_12_).^10^ In contrast to the large number of borophosphates, borate phosphates are rare, particularly those that exclusively exhibit tetrahedral borate and phosphate groups such as the mineral seamanite Mn_3_(OH)_2_[B(OH)_4_][PO_4_].^11^ Th_2_[BO_4_][PO_4_]^12^ and the barium uranyl borate phosphate Ba_5_[(UO_2_)(PO_4_)_3_(B_5_O_9_)]**·**H_2_O^13^ are the only two actinide borate phosphates. Whereas Th_2_[BO_4_][PO_4_] contains isolated [BO_4_]^5–^ and [PO_4_]^3–^ groups, the barium uranyl borate phosphate is based on phosphate groups linked to the uranyl polyhedra, which are further connected to borate tubes. The understanding of the chemistry of actinide borates and phosphates has an urgent importance in the question of the storage of nuclear waste. Owing to the high stability and insolubility of these compounds, they are of interest for the immobilization of nuclear waste. For example, Wang et al. reported a thorium borate with the composition [ThB_5_O_6_(OH)_6_][BO(OH)_2_]**·**2.5H_2_O,^14^ which possesses disordered borate tetrahedra in the channels of the structure that can be exchanged by (TcO_4_)^–^ groups. The topicality of this research in actinide borates is demonstrated by work recently published by Wu et al.^15^ Here, the application of high-pressure/high-temperature conditions enabled the synthesis of a uranium borate phosphate with the composition U_2_[BO_4_][PO_4_], which is isotypic to the recently discovered Th_2_[BO_4_][PO_4_].^12^ We describe the synthesis of U_2_[BO_4_][PO_4_], its single-crystal structure determination, Raman spectroscopic investigations, and a comparison to the isotypic phase Th_2_[BO_4_][PO_4_].

## Results and Discussion

### Synthesis and Crystal Structure Analysis

U_2_[BO_4_][PO_4_] was synthesized from UO_3_, H_3_BO_3_, and P_4_O_10_ under high-pressure/high-temperature conditions of 12.5 GPa and 1000 °C in a 1000 ton multianvil press with a Walker-type module. A detailed description of the synthesis is provided in the Experimental Section. The single-crystal intensity data were collected at room temperature with a Nonius KappaCCD diffractometer with graphite-monochromated Mo-*K_α_* radiation (*λ* = 71.073 pm). In contrast to the structural refinement of the isotypic Th_2_[BO_4_][PO_4_] phase by Lipp et al.,^12^ we found split positions of the uranium atoms with a ratio of 0.93(1):0.07(1) for both sites of the heavy atoms U1a/U1b and U2a/U2b. The reason for the presence of these split positions is presumably a boron/phosphorus disorder at the tetrahedral centers of the [BO_4_]^5–^ and [PO_4_]^3–^ groups. The omission of the refinement of split positions led to significant residual peaks near the uranium atoms and to the impossibility to calculate the anisotropic atomic displacement parameters of the boron atoms. The single-crystal measurement of a second single crystal led to the same disorder with the same ratio. It was impossible to refine the oxygen atoms with split positions; therefore, the minor part of the split uranium atoms was not described with a similar coordination environment as the major part. Therefore, the calculation of the coordination spheres of U1b, U2b, P1b, and B1b was not practicable. Owing to the high standard deviations of the boron–oxygen distances in the isotypic Th[BO_4_][PO_4_] phase, we assume a similar disorder in that structure. Hence, the structure description of U_2_[BO_4_][PO_4_] was performed on the basis of the main split positions U1a and U2a, which are denoted as U1 and U2 respectively, in the following. Tables 1, 2, and 7 list details of the data collection and evaluation as well as the positional parameters of the refinement. Interatomic distances and angles are listed in Tables 3 and 4.

**Table 1:** Atomic coordinates and equivalent isotropic displacement parameters *U*_eq_ [Å^2^] of U_2_[BO_4_][PO_4_] (space group: *P*2_1_/*c*). All atoms are positioned on the Wyckoff site 8*c*. *U*_eq_ is defined as one third of the trace of the orthogonalized *U_ij_* tensor (standard deviations in parentheses).

Atom	*x*/*a*	*y*/*b*	*z*/*c*	*U*_eq_	Site occupancy
U1a	0.69822(8)	0.30549(5)	0.05204(6)	0.0155(2)	0.93(1)
U1b	0.756(2)	0.304(2)	0.0860(9)	0.019(2)	0.07(1)
U2a	0.26598(6)	0.18644(5)	0.07935(6)	0.0078(2)	0.93(1)
U2b	0.222(2)	0.194(2)	0.043(2)	0.049(4)	0.07(1)
B1a	0.4620(9)	0.4475(9)	0.2813(9)	0.006(2)	0.93(1)
B1b	0.0451(3)	0.5404(3)	0.2188(3)	0.0064(4)	0.07(1)
P1a	0.0451(3)	0.5404(3)	0.2188(3)	0.0064(4)	0.93(1)
P1b	0.4620(9)	0.4475(9)	0.2813(9)	0.005(2)	0.07(1)
O1	0.5551(7)	0.1135(7)	0.1412(8)	0.008(2)	1.00
O2	0.4002(8)	0.4511(8)	0.0968(7)	0.010(2)	1.00
O3	0.3814(8)	0.3011(8)	0.3446(8)	0.011(2)	1.00
O4	0.6381(7)	0.4139(8)	0.3163(8)	0.008(2)	1.00
O5	0.1349(7)	0.6885(8)	0.1598(8)	0.011(2)	1.00
O6	0.0654(9)	0.3699(8)	0.1358(9)	0.015(2)	1.00
O7	0.1177(8)	0.5347(8)	0.4094(8)	0.011(2)	1.00
O8	0.1380(7)	0.0731(8)	0.3162(8)	0.010(2)	1.00

**Table 2:** Anisotropic displacement parameters *U_ij_* [Å^2^] for U_2_[BO_4_][PO_4_] (space group: *P*2_1_/*c*).

Atom	*U*_11_	*U*_22_	*U*_33_	*U*_12_	*U*_13_	*U*_23_
U1a	0.0298(3)	0.0056(2)	0.0136(2)	–0.0010(2)	0.0102(2)	0.0009(2)
U2a	0.0102(2)	0.0056(2)	0.0077(2)	–0.0008(2)	0.0025(2)	–0.0010(2)
B1a	0.004(3)	0.005(3)	0.009(3)	–0.001(2)	0.003(2)	0.001(2)
B1b	0.006(2)	0.0065(9)	0.0066(9)	–0.0017(7)	0.0018(8)	–0.0002(7)
P1a	0.006(2)	0.0065(9)	0.0066(9)	–0.0017(7)	0.0018(8)	–0.0002(7)
P1b	0.004(3)	0.005(3)	0.009(3)	–0.001(2)	0.003(2)	0.001(2)
O1	0.007(3)	0.008(3)	0.010(3)	0.001(2)	0.002(2)	0.002(2)
O2	0.014(3)	0.006(3)	0.007(3)	–0.004(2)	0.000(2)	0.000(2)
O3	0.013(3)	0.010(3)	0.014(3)	–0.004(2)	0.009(2)	0.000(2)
O4	0.005(3)	0.004(2)	0.013(3)	–0.004(2)	0.001(2)	–0.002(2)
O5	0.014(3)	0.008(3)	0.012(3)	–0.004(2)	0.002(2)	0.001(2)
O6	0.024(4)	0.008(3)	0.014(3)	0.002(2)	0.006(3)	–0.002(2)
O7	0.013(3)	0.007(3)	0.011(3)	0.002(2)	0.003(2)	0.000(2)
O8	0.007(3)	0.007(3)	0.016(3)	0.001(2)	0.001(2)	0.002(2)

**Table 3:** Interatomic distances [pm] in U_2_[BO_4_][PO_4_] (space group: *P*2_1_/*c*), calculated from the single-crystal lattice parameters.

U1–O1	215.1(6)	U2–O3	234.0(6)
U1–O2	230.3(6)	U2–O2	234.0(6)
U1–O5	246.9(6)	U2–O6	234.8(7)
U1–O4	247.0(6)	U2–O3′	235.1(6)
U1–O4′	253.6(6)	U2–O4	235.8(6)
U1–O8	260.2(6)	U2–O7	238.9(6)
U1–O7	260.2(6)	U2–O1	247.7(6)
U1–O5′	263.3(6)	U2–O8	257.3(6)
U1–O2′	288.0(6)	U2–O8′	287.5(6)
U1–O3	298.4(2)		
	av. 256.3		av. 245.0
P1–O6	151.3(7)	B1–O1	145.5(9)
P1–O5	151.7(6)	B1–O3	147.8(9)
P1–O7	154.6(7)	B1–O2	148.4(9)
P1–O8	154.9(7)	B1–O4	149.2(9)
	av. 153.1		av. 147.7

**Table 4:** Interatomic angles [°] in U_2_[BO_4_][PO_4_] (space group *P*2_1_/*c*), calculated from the single-crystal lattice parameters.

O1–B1–O3	115.4(6)	O6–P1–O5	113.8(4)
O1–B1–O2	112.2(6)	O6–P1–O7	111.5(4)
O3–B1–O2	106.2(6)	O5–P1–O7	102.8(4)
O1–B1–O4	105.3(6)	O6–P1–O8	105.7(4)
O3–B1–O4	109.0(6)	O5–P1–O8	112.2(4)
O2–B1–O4	108.6(6)	O7–P1–O8	111.0(4)
	av. 109.5		av. 109.5

### Crystal Structure

**Figure 1:**
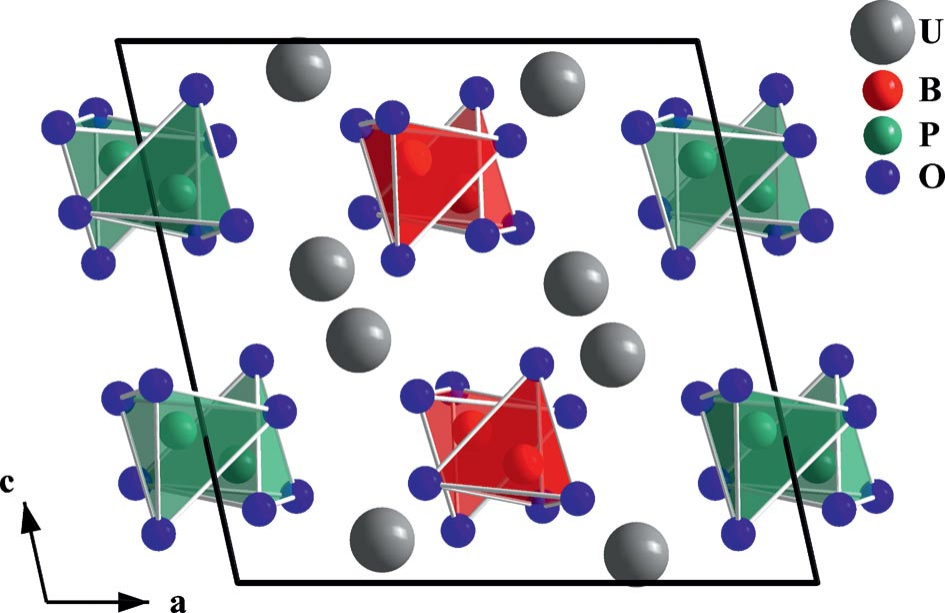
Crystal structure of the new actinide borate phosphate U_2_[BO_4_][PO_4_] (space group: *P*2_1_/*c*) down [010], which shows isolated [BO_4_^5–^] and [PO_4_^3–^] groups.

**Figure 2:**
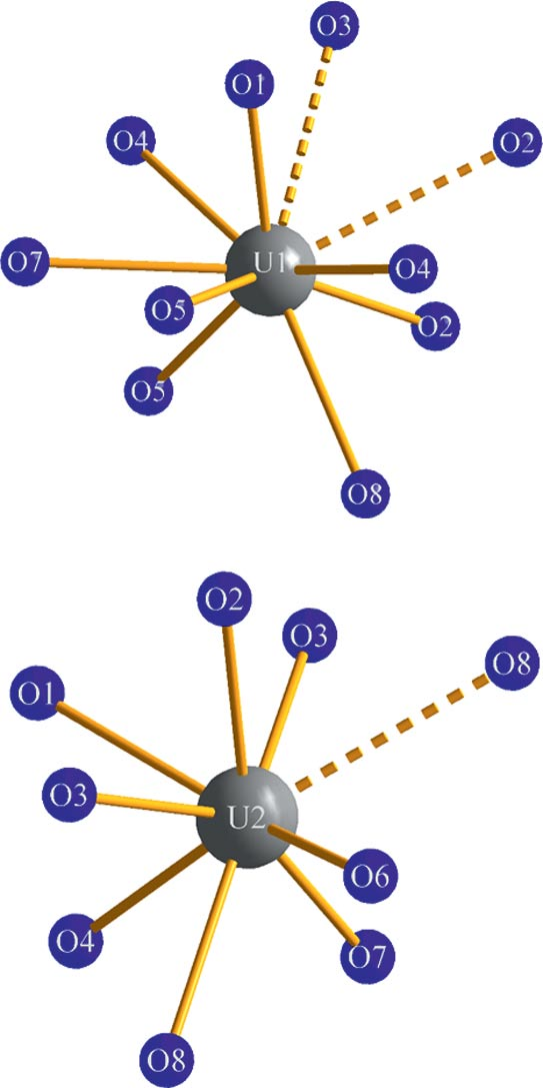
Coordination spheres of the U^4+^ ions in U_2_[BO_4_][PO_4_]. The U1^4+^ cation is surrounded by ten oxygen anions, and U2^4+^ is surrounded by nine oxygen anions to form [U(1)O_10_]^16–^ and [U(2)O_9_]^14–^ polyhedra, respectively.

The new actinide borate phosphate U_2_[BO_4_][PO_4_] crystallizes in the monoclinic space group *P*2_1_/*c* with four formula units per unit cell and the lattice parameters *a* = 854.6(2), *b* = 775.3(2), *c* = 816.3(2) pm, and *β* = 102.52(3)°. The structure consists of two crystallographically different tetravalent uranium cations and isolated tetrahedral [BO_4_]^5–^ and [PO_4_]^3–^ groups. Figure 1 gives a view of the crystal structure along [010], which shows the isolated tetrahedra. From the formula U_2_[BO_4_][PO_4_], the valence of the uranium cations calculates to 4+ owing to the principle of electroneutrality. This is confirmed by the intense emerald green color of the crystals and additionally verified by calculations of the bond valence sums by using the bond length/bond strength (Σ*V*)^16^,^17^ and the charge distribution in solids concept (CHARDI, Σ*Q*).^18^ The results of these calculations for all atoms are listed in Table 5. The U1 cations are coordinated by ten oxygen atoms to build up [U(1)O_10_]^16–^ polyhedra, and the U2 cations are coordinated by nine oxygen atoms to form [U(2)O_9_]^14–^ polyhedra. From the longest uranium–oxygen distances [U1–O3 298.4(2) pm and U2–O3 287.5(6) pm] and the large difference to the third longest U1–O distance [U1–O7 263.3(6) pm] and the second longest U2–O distance [U2–O7 257.3(6) pm], the description as an 8+2 coordination for U1 and an 8+1 coordination for U2 is reasonable. Calculations of the effective coordination number (*δ*-ECoN) values by using the program MAPLE (madelung part of lattice energy)^19^–^21^ resulted in a value of 0.09 for O3 [U1–O3 298.4(2) pm] in the coordination sphere of U1. Owing to the low value, the coordination sphere of U1 can also be described as an 8+1 coordination; however, following the structure description of Th_2_[BO_4_][PO_4_],^12^ we describe the U1 atom with a tenfold (8+2) coordination. These coordination spheres result in uranium–oxygen distances between 215.2(6) and 298.4(2) pm with a mean value of 256.3 pm for U1 and between 234.0(6) and 287.5(6) pm with a mean value of 245.0 pm for U2. Figure 2 displays the coordination spheres of the uranium ions.

**Table 5:** Charge distribution in U_2_[BO_4_][PO_4_] (space group *P*2_1_/*c*), calculated from the bond length/bond strength (Σ*V*) and the CHARDI (Σ*Q*) concept.

	U1	U2	B1	P1	O1	O2
Σ*V*	+3.47	+3.90	+3.00	+4.88	–2.07	–2.00
Σ*Q*	+3.83	+4.08	+2.90	+5.20	–2.26	–2.11
	O3	O4	O5	O6	O7	O8
Σ*V*	–1.81	–1.93	–1.89	–1.81	–1.91	–1.84
Σ*Q*	–1.85	–2.09	–2.05	–1.89	–1.96	–1.79

**Figure 3:**
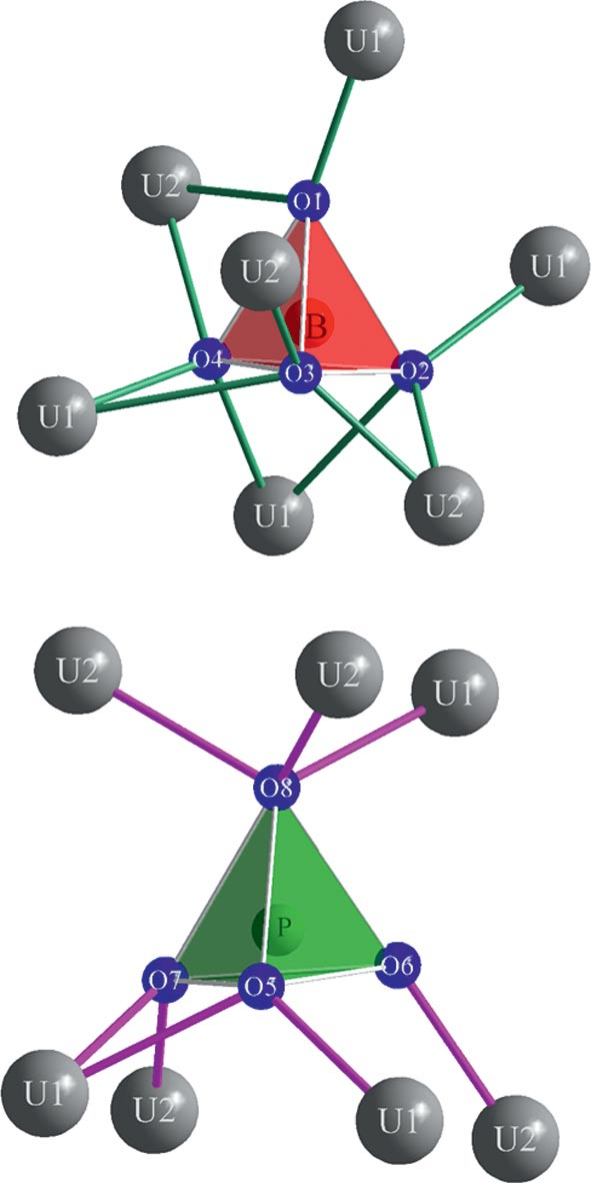
Borate and phosphate tetrahedra in the structure of U_2_[BO_4_][PO_4_] (space group: *P*2_1_/*c*) and their neighboring uranium cations. The [BO_4_]^5–^ groups shares four edges with the uranium polyhedra, whereas the [PO_4_]^3–^ groups possess only one common edge with a uranium–oxygen polyhedron.

**Figure 4:**
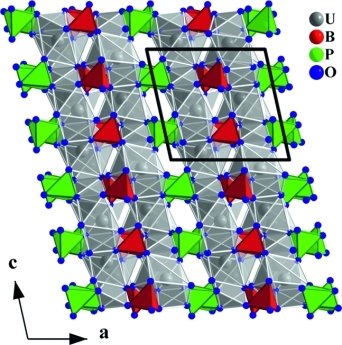
Crystal structure of U_2_[BO_4_][PO_4_] down [010], which shows layers built up from linked uranium–oxygen polyhedra.

The crystal structure contains one boron atom that is tetrahedrally coordinated by four oxygen atoms. The boron–oxygen distances of the tetrahedra vary between 145.5(9) (B1–O1) and 149.3(9) pm (B1–O4) with a mean value of 147.7 pm. This value agrees well with the known average value for B–O distances in [BO_4_]^5–^ groups (147.6 pm).^22^–^24^ The oxygen–boron–oxygen angles of the tetrahedral [BO_4_]^5–^ groups are listed in Table 4 and correspond well to the expected angles of tetrahedral groups. The crystallographically distinct phosphate site is coordinated by four oxygen atoms with P–O bond lengths from 151.3(7) (P1–O6) to 154.9(7) pm (P1–O8) with a mean value of 153.1 pm. These values agree well with those of isolated phosphate groups found in other uranium phosphates such as U_2_(PO)_4_(P_3_O_10_) (P–O 151.2–157.6 pm),^5^ rare-earth borate phosphates such as *RE*_7_O_6_(BO_3_)(PO_4_)_2_ (*RE* = Pr, Sm; P–O 151.0–154.7 pm),^25^ or the average value in phosphate minerals (153.7 pm).^26^ The [BO_4_]^5–^ groups share four edges with the neighboring uranium–oxygen polyhedra, whereas the [PO_4_]^3–^ anions have only one single edge with the uranium–oxygen polyhedra. Figure 3 shows the borate and phosphate tetrahedra in U_2_[BO_4_][PO_4_] and their adjacent U^4+^ cations. The [(U1)O_10_]^16–^ and [(U2)O_9_]^14–^ polyhedra form chains along [001] by sharing edges with each of the neighboring polyhedra of the same kind. These chains build sheets in the *bc* plane by sharing common edges. Two of these sheets are linked to a double layer, which is also in the *bc* plane. The tetrahedral [BO_4_]^5–^ groups are located between the layers inside of the double layer. The phosphate groups link the double layers. Figure 4 illustrates the double layers and the position of the tetrahedral borate and phosphate groups in the crystal structure of U_2_[BO_4_][PO_4_]. For a more detailed description of the structure, the reader is referred to the description of the isotypic compound Th_2_[BO_4_][PO_4_].^12^ Table 6 gives a comparison of the unit cells, the coordination numbers of the actinide metal ions, and the bond lengths of U_2_[BO_4_][PO_4_] and the isotypic Th_2_[BO_4_][PO_4_]. A closer look at the lattice parameters *a*, *b*, *c*, and *β* reveals a prominent decrease of the lattice parameter *b* (–2.6 %) in contrast to the lattice parameters *a* (+0.9 %) and *c* (–0.8 %). This divergence emerges from the higher ionic radius of Th^4+^ and the positioning of the actinide cations in the *bc* plane. The actinide contraction leads to a shrinkage of the cation layers associated with a decreases of the lattice parameters *b* and *c*. Furthermore, the shrinkage of the cation layers in the *bc* plane leads to shorter actinide–actinide distances and additionally to an enlargement of the distances of the tetrahedral borate and phosphate groups along *a* and, therefore, an increase of the lattice parameter *a*. The coordination numbers of the actinide atoms (U, Th) are equivalent. As expected, the *An*–O (*An* = U, Th) distances in Th_2_[BO_4_][PO_4_] are larger because of the larger ionic radius of Th^4+^. A comparison of the tetrahedral borate and phosphate groups shows no greater deviances of the bond lengths and angles.

**Table 6:** Comparison of the isotypic structures U_2_[BO_4_][PO_4_] and Th_2_[BO_4_][PO_4_]^12^ (both space group *P*2_1_/*c*).

Empirical formula	U_2_[BO_4_][PO_4_]	Th_2_[BO_4_][PO_4_]
*a* /pm	854.6(2)	846.65(2)
*b* /pm	775.3(2)	795.52(2)
*c* /pm	816.3(2)	822.97(1)
*β* /°	102.52(3)	103.75(1)
*An*1 (*An* = U, Th) coordination number	10	10
*An*2 (*An* = U, Th) coordination number	9	9
Av. *An*1–O (*An* = U, Th) distance /pm	251.7	259.1
Av. *An*2–O (*An* =U, Th) distance /pm	245.0	248.6
Av. P–O distance in [PO_4_]^3–^ groups /pm	153.1	152.9
Av. B–O distance in [BO_4_]^5–^ groups /pm	147.7	147.1

### Raman Spectroscopy

**Figure 5:**
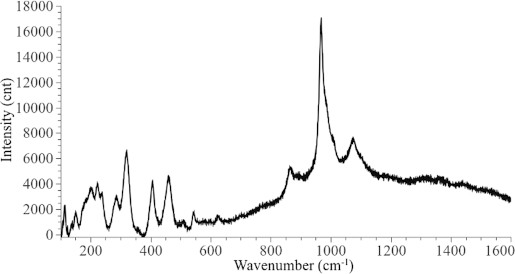
Raman spectrum of a single crystal of U_2_[BO_4_][PO_4_] (space group: *P*2_1_/*c*) in the range 100–1600 cm^–1^.

Figure 5 shows the Raman spectrum of U_2_[BO_4_][PO_4_] from 100 to 1600 cm^–1^. In the range 3000 to 3600 cm^–1^, no OH or water bands could be detected. Bands at ca. 900 cm^–1^ in oxoborates are usually assigned to stretching modes of the [BO_4_]^5–^ groups.^27^–^30^ Bands at wavenumbers smaller than 500 cm^–1^ can be assigned to U–O bonds as well as lattice vibrations. Bands between 1000 and 1100 cm^–1^ refer to the ν_1_(PO_4_)^3–^ symmetric stretching mode and the ν_3_(PO_4_)^3–^ antisymmetric stretching mode.^31^

## Conclusions

With the successful synthesis of U_2_[BO_4_][PO_4_], the first isotypic compound to Th_2_[BO_4_][PO_4_] was found and characterized. The structure is built up by isolated [BO_4_]^5–^ and [PO_4_]^3–^ groups and double layers formed by linked uranium–oxygen polyhedra. The application of similar synthetic conditions to other tetravalent cations with similar ionic radii, such as Ce^4+^, could lead to additional isotypic compounds and will be studied in the future.

## Experimental Section

***Caution:*** Working with UO_3_ requires precautions for the handling of radioactive and toxic substances.

**Synthesis:** The uranium borate phosphate U_2_[BO_4_][PO_4_] was synthesized by a two-stage synthesis. The synthesis of the precursor was achieved under high-pressure/high-temperature conditions of 7.0 GPa and 700 °C from a nonstoichiometric mixture of UO_3_ [46.63 mg, synthesized by pyrolysis of UO_2_(NO_3_)_2_**·**6H_2_O at 300 °C], H_3_BO_3_ (30.13 mg; Carl Roth GmbH + Co. KG, Karlsruhe, Germany, 99.8+%), and partially hydrolyzed P_4_O_10_ (23.14 mg; Merck GmbH, Darmstadt, Germany, p.a.). The starting materials were finely ground, inserted into a gold capsule, and placed in a boron nitride crucible (Henze BNP GmbH, HeBoSint® S100, Kempten, Germany). The boron nitride crucible was placed into an 18/11-assembly and compressed by eight tungsten carbide cubes (TSM-10, Ceratizit, Reutte, Austria). To apply the pressure, a 1000 ton multianvil press with a Walker-type module (both devices from Voggenreiter, Mainleus, Germany) was used. A detailed description of the assembly preparation can be found in refs.^32^–^36^ In detail, the 18/11-assembly was compressed to 7.0 GPa in 200 min and heated to 700 °C (cylindrical graphite furnace) in the following 10 min, kept there for 10 min, and cooled down to 300 °C in 30 min at constant pressure. After natural cooling to room temperature by switching off the heating, a decompression period of 10 h was required. The recovered MgO octahedron (pressure transmitting medium, Ceramic Substrates & Components Ltd., Newport, Isle of Wight, UK) was broken apart, and the sample was carefully separated from the surrounding graphite and boron nitride crucible. The precursor was gained in the form of a yellow tough bulk. The bulk was dried for 3 h in a compartment dryer at 80 °C. The X-ray powder diffraction analysis showed an amorphous phase. The yellow color might indicate a hexavalent uranium compound. This amorphous precursor was the starting material for the synthesis of U_2_[BO_4_][PO_4_]. For the synthesis, high-pressure/high-temperature conditions of 12.5 GPa and 1000 °C were required by using an 14/11-assembly. The preparation of the assembly was equal to the preparation described before. In detail, the 14/11 assembly was compressed to 12.5 GPa in 380 min and heated to 1000 °C (cylindrical graphite furnace) in the following 10 min, kept there for 5 min, and cooled to 450 °C in 25 min at constant pressure. After natural cooling to room temperature by switching off the heating, a decompression period of 12 h was required. The new compound U_2_[BO_4_][PO_4_] was gained in form of emerald green, air- and water-resistant crystals in an emerald green amorphous matrix.

**X-ray Powder Diffraction:** The powder diffraction pattern of U_2_[BO_4_][PO_4_] was obtained in transmission geometry from flat samples of the reaction product by using a STOE STADI P powder diffractometer with Mo-*K_α_*_1_ radiation (Ge monochromator, *λ* = 70.93 pm). In addition to the diffraction pattern of U_2_[BO_4_][PO_4_], we found reflections of a still-unknown side product and an amorphous phase. The experimental powder pattern tallies well with the theoretical pattern simulated from single-crystal data.

**X-ray Structure Determination:** Small single crystals of the new actinide borate phosphate U_2_[BO_4_][PO_4_] could be isolated by mechanical fragmentation. The single-crystal intensity data of U_2_[BO_4_][PO_4_] were collected at room temperature by using a Nonius KappaCCD diffractometer with graphite-monochromated Mo-*K_α_* radiation (*λ* = 71.073 pm). A semiempirical absorption correction based on equivalent and redundant intensities (Scalepack)^37^ was applied to the intensity data. All relevant details of the data collection and evaluation are listed in Table 7.

**Table 7:** Crystal data and structure refinement of U_2_[BO_4_][PO_4_] (space group *P*2_1_/*c*, standard deviations in parentheses).

Empirical formula	U_2_[BO_4_][PO_4_]
Molar mass /g mol^–1^	645.84
Crystal system	monoclinic
Space group	*P*2_1_/*c* (No. 14)
Diffractometer	Enraf–Nonius KappaCCD
Radiation	Mo-*K_α_* (*λ* = 71.073 pm)
*a* /pm	854.6(2)
*b* /pm	775.3(2)
*c* /pm	816.3(2)
*β* /°	102.52(3)
*V* /Å^3^	528.0(2)
Formula units per cell	4
Calculated density /g cm^–3^	8.12
Crystal size /mm^3^	0.04 × 0.04 × 0.03
Temperature, *K*	293(2)
Absorption coefficient /mm^–1^	61.6
*F*(000)	1072
*θ* range /°	2.4–32.5
Range in *hkl*	±12, ±11, ±12
Total no. of reflections	7336
Independent reflections	1911 (*R*_int_ = 0.0717)
Reflections with *I* ≥ 2σ(*I*)	1607 (*R*_σ_ = 0.0470)
Data/parameters	1911/118
Absorption correction	multiscan (Scalepack^37^)
Goodness-of-fit on *F*_i_^2^	1.133
Final *R* indices [*I* ≥ 2σ(*I*)]	*R*_1_ = 0.0388
	*wR*_2_ = 0.0658
*R* indices (all data)	*R*_1_ = 0.0531
	*wR*_2_ = 0.0690
Largest diff. peak and hole /e Å^–3^	4.27/–1.98

According to the systematic extinctions, the monoclinic space group *P*2_1_/*c* was derived. The structure solution and parameter refinement (full-matrix least-squares against *F*^2^) were performed by using the SHELX-97 software suite^38^,^39^ with anisotropic atomic displacement parameters for the atoms of the main split position. The final difference Fourier syntheses did not reveal any significant residual peaks in all refinements. The ratio of the occupation disorder was confirmed by two methods. First, the refinement with a free variable *x* for the major part and (1 – *x*) for the minor part was done automatically by the program and converged to a value of 0.93. Second, the manual variation of the occupation factors to equal isotropic displacement parameters for the positions at P1a and B1a. This method works with the simple assumption of a nearly equal mobility of the phosphate and borate anions in the crystal lattice. Therefore, the anisotropic displacement parameters of the phosphorus and boron atoms were reset to isotropic values and the refinement astonishingly leads to the same occupation factor of 0.93 for P1a and B1a. The positional parameters of the refinements, anisotropic displacement parameters, interatomic distances, and interatomic angles are listed in the Table 1, Table 2, Table 3, and Table 4.

Further details on the crystal structure investigations may be obtained from the Fachinformationszentrum Karlsruhe, 76344 Eggenstein-Leopoldshafen, Germany (http://www.fiz-informationsdienste.de/en/DB/icsd/depotanforderung.html; crysdata@fiz-karlsruhe.de; fax: +49-7247-808-666), on quoting the depository number CSD-426154.

**Vibrational Spectroscopy:** The confocal Raman spectra of the single crystals of U_2_[BO_4_][PO_4_] in the range 50–4000 cm^–1^ were recorded with a Horiba Jobin Yvon Labram-HR 800 Raman microspectrometer. The samples were excited by using the 532 nm emission line of a frequency-doubled 100 mW Nd:YAG laser and the 633 nm emission line of a 17 mW helium neon laser under an Olympus 50 × objective lens. The size of the laser spot on the surface was approximately 1 μm. The scattered light was dispersed by an optical grating with 1800 lines mm^–1^ and collected by a 1024 × 256 open electrode CCD detector. The spectral resolution, determined by measuring the Rayleigh line, was less than 2 cm^–1^. The spectra were recorded unpolarized. The accuracy of the Raman line shifts, calibrated by regularly measuring the Rayleigh line, was in the order of 0.5 cm^–1^. Background and Raman bands were fitted by the built-in spectrometer software LabSpec to second-order polynomial and convoluted Gaussian–Lorentzian functions, respectively.

## References

[1] Weil M, Glaum R (2000). Acta Crystallogr., Sect. C.

[2] Panagiotidis K, Glaum R (2008). Acta Crystallogr., Sect. E.

[3] Gasperin M (1987). Acta Crystallogr., Sect. C.

[4] Wang S, Alekseev EV, Stritzinger JT, Depmeier W, Albrecht-Schmitt TE (2010). Inorg. Chem.

[5] Höppe HA, Daub M (2012). Z. Kristallogr.

[6] Cabeza A, Aranda MAG, Cantero FM, Lozano D, Martinez-Lara M, Bruque S (1996). J. Solid State Chem.

[7] Podor R, Francois M, Dacheux N (2003). J. Solid State Chem.

[8] Huang Y, Ewald B, Schnelle W, Prots Y, Kniep R (2006). Inorg. Chem.

[9] Sen Gupa PK, Swihart GH, Dimitrijevic R, Hossain MB (1991). Am. Mineral.

[10] Kniep R, Gözel G, Eisenmann B, Röhr C, Asbrand M, Kizilyalli M (1994). Angew. Chem.

[11] Moore PB, Ghose S (1971). Am. Mineral.

[12] Lipp C, Burns PC (2011). Can. Mineral.

[13] Wu S, Wang S, Diwu J, Depmeier W, Malcherek T, Alekseev EV, Albrecht-Schmitt TE (2012). Chem. Commun.

[14] Wang S, Alekseev EV, Juan D, Casey WH, Phillips BL, Depmeier W, Albrecht-Schmitt TE (2010). Angew. Chem. Int. Ed.

[15] Wu S, Wang S, Polinski M, Beerbaum O, Kegler P, Malcherek T, Holzheid A, Depmeier W, Bosbach D, Albrecht-Schmitt TE, Alekseev EV (2013). Inorg. Chem.

[16] Brown ID, Altermatt D (1985). Acta Crystallogr., Sect. B.

[17] Brese NE, O'Keeffe M (1991). Acta Crystallogr., Sect. B.

[18] Hoppe R, Voigt S, Glaum H, Kissel J, Müller HP, Bernet KJ (1989). J. Less-Common Met.

[19] Hoppe R (1966). Angew. Chem.

[20] Hoppe R (1970). Angew. Chem.

[21] R. Hübenthal, *MAPLE - Program for the Calculation of MAPLE Values*

[22] Zobetz E (1990). Z. Kristallogr.

[23] Hawthorne FC, Burns PC, Grice JD, Grew ES (1996). Boron: Mineralogy, Petrology and Geochemistry.

[24] Zobetz E (1982). Z. Kristallogr.

[25] Ewald B, Prots Y, Kniep R (2004). Z. Kristallogr.

[26] Huminicki DMC, Hawthorne FC (2002). Rev. Mineral. Geochem.

[27] Hinteregger E, Heymann G, Hofer TS, Huppertz H (2012). Z. Naturforsch. B.

[28] Pitscheider A, Kaindl R, Oeckler O, Huppertz H (2011). J. Solid State Chem.

[29] Ren M, Lin JH, Dong Y, Yang LQ, Su MZ, You LP (1999). Chem. Mater.

[30] Laperches JP, Tarte P (1966). Spectrochim. Acta.

[31] Frost RL, Čejka J, Ayoko G (2008). J. Raman Spectrosc.

[32] Kawai N, Endo S (1970). Rev. Sci. Instrum.

[33] Walker D, Carpenter MA, Hitch CM (1990). Am. Mineral.

[34] Walker D (1991). Am. Mineral.

[35] Rubie DC (1999). Phase Transitions.

[36] Huppertz H (2004). Z. Kristallogr.

[37] Otwinowski Z, Minor W (1997). Methods Enzymol.

[38] G. M. Sheldrick, *SHELXS-97**and**SHELXL-97**Program Suite for the Solution and Refinement of Crystal Structures*

[39] Sheldrick GM (2008). Acta Crystallogr., Sect. A.

